# Association between MPO-463G > A polymorphism and chronic kidney disease: a meta-analysis

**DOI:** 10.1080/0886022X.2018.1499529

**Published:** 2018-10-03

**Authors:** Jiaxuan Qin, Jinchun Xing, Wei Li, Kaiyan Zhang, Zhun Wu

**Affiliations:** a Department of Urology Surgery, The First Affiliated Hospital of Xiamen University, Xiamen, Fujian, China;; b Center of Diagnosis and Treatment of Urinary System Diseases, The First Affiliated Hospital of Xiamen UniversityXiamen, Fujian, China;; c The Key Laboratory of Urinary Tract Tumors and Calculi of Xiamen City, The First Affiliated Hospital of Xiamen University, Xiamen, Fujian, China

**Keywords:** Myeloperoxidase, single nucleotide polymorphism, rs2333227, chronic kidney disease, meta-analysis

## Abstract

**Background/objective*:*** Previous studies have shown that MPO -463G > A (rs2333227) might be associated with chronic kidney disease (CKD) susceptibility, but sample sizes of those studies are relatively small. Hence, we decided to perform a meta-analysis to evaluate the association.

**Methods/main results:** Two investigators search databases systematically and independently. Odds ratios and 95% confidence intervals were used to pool the effect size. Four articles with 618 cases and 932 controls in total were included in our meta-analysis.

**Conclusions:** MPO -463G > A was not associated with CKD susceptibility in recessive model and homozygote comparison. MPO -463G > A was associated with increased risk of CKD in allelic comparison, heterozygote comparison and dominant model, however, the results lacked stability. Owing to insufficient data, the association between MPO -463G > A and CKD cannot be fully confirmed.

## 1. Introduction

Myeloperoxidase (MPO) is an oxidative lysosomal enzyme that is available in polymorphonuclear neutrophils and monocytes. MPO utilizes H_2_O_2_ to generate hypochlorous acid (HClO) and other reactive moieties, which kill pathogens during infections. In contrast, in the setting of sterile inflammation, MPO and MPO-derived oxidants are thought to be pathogenic, promoting inflammation and causing tissue damage [1]. Patients with chronic kidney disease (CKD) have a number of disorders in the organism. Chronic inflammation joined with oxidative stress contributes to the development of numerous complications: accelerated atherosclerosis process and cardiovascular disease, emergence of type 2 diabetes mellitus, development of malnutrition, anemia, hyperparathyroidism, and so forth, affecting the prognosis and quality of life of patients with CKD [2]. Peripheral blood myeloperoxidase activity increases during hemodialysis [3].

MPO -463G > A (rs2333227) is a single nucleotide polymorphism (SNP) in position -463 of MPO gene’s 5′ upstream region. The -463 G creates a stronger SP1 binding site, which can increase MPO expression than -463 A [4].

Previous studies have shown that MPO -463G > A (rs2333227) might be associated with chronic kidney disease (CKD) susceptibility, but sample sizes of those studies are relatively small. Hence, we conducted a meta-analysis to evaluate the association.

## 2. Methods and materials

### 2.1. Eligible study identification

Without any limitation, two investigators used the following terms to search databases systematically and independently: ’myeloperoxidase or MPO’ and ‘failure or dialysis or injury or ESRD or nephropathy or chronic kidney disease or CKD or end-stage’ and ‘renal or kidney’ and ‘polymorphisms or polymorphism’. PubMed, Embase, Cochrane Library, clinicaltrials.gov, and CNKI databases were searched up to July 26, 2017. We also searched the references of related reviews and studies manually.

### 2.2. Inclusion and exclusion criteria

Inclusion criteria of this meta-analysis: (1) case-control study about the association between MPO -463G > A polymorphism and human chronic kidney disease; (2) enough genotype data. Exclusion criteria of this meta-analysis: (1) repetitive study (only the study with the largest population was included); (2) lack of enough genotype data; (3) editorial, comment, and review; (4) Genome Wide Association Study; (5) studies in cell lines. Academic dissertation was also reviewed. We try to get detailed genotype data by emailing the author.

On the basis of inclusion and exclusion criteria above, two investigators selected studies independently and the investigators resolved divergence by discussion.

### 2.3. Data extraction of eligible studies

Data were extracted by two investigators independently. The investigators resolve divergence by discussion. The information below were extracted: first author’s name, publication year, nephropathy type, cases’ and controls’ characteristics, control groups’ source, country, ethnicity, sample for detection, genotyping method, Hardy–Weinberg equilibrium, number of cases and controls for each genotype.

### 2.4. Methodological quality assessment

On the basis of Newcastle–Ottawa Scale (NOS) [5], two investigators independently evaluated the qualities of eligible studies and ‘age, gender and country’ were set as the most important factor. Quality scores range from 0 to 9, and better quality with higher scores. The investigators resolve divergence by discussion.

### 2.5. Statistics analysis

On the basis of the PRISMA checklists [6], our meta-analysis was conducted. By Chi-square test, control groups’ Hardy–Weinberg equilibrium (HWE) was evaluated for each study, and the significant departure from HWE is *p* < .05. To assess the strength of the association between MPO -463G > A polymorphism and chronic kidney disease susceptibility, OR and 95% CIs were counted. We got pooled ORs grom respective combination of single studies by allelic comparison (A vs. G), dominant model (GA + AA vs. GG), recessive model (AA vs. GG + GA), homozygote comparison (AA vs. GG) and heterozygote comparison (GA vs. GG). Z-test with *p* values less than .05 means statistical significant level.

Q-test and I^2^ index were used to assess heterogeneity [7]. The random-effects model (DerSimonian and Laird method) was used when Q-test’s *p* values was less than .10 and/or I^2^ index was more than 50%; otherwise, we performed fixed-effects model (Mantel and Haenszel method) [8]. To assess the effect of each study on combined ORs, sensitivity analyses were conducted towards each genetic model by sequentially excluding each study in total and in any subgroup including more than two studies. Moreover, subgroup analyses were stratified by nephropathy type. The publication bias was evaluated by using Begg’s funnel [9] plot and Egger’s test [10] in every genetic model. An asymmetric plot, the *p* values of Begg’s test (P_B_) less than .05, and the *p* values of Egger’s test (P_E_) less than .05 means a significant publication bias. We did all statistical analyses by using Stata 12.0 software (StataCorp, College Station, Texas, USA). Except for specified conditions, two-tailed *p* < .05 means significant. Further statistics analysis was done in allelic comparison (A vs. G) with XLSTAT 2014.4.04 software (Copyright Addinsoft 1995–2014), in which a kind of logistic regression called Correlated Component Regression was used.

## 3. Results

### 3.1. Studies’ characteristics

**Figure 1.**
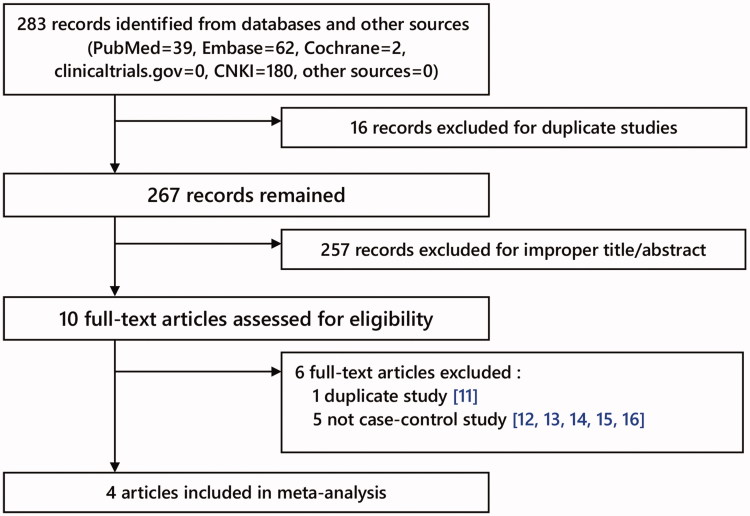
Flow Chart of study selection.

In total, we obtained 283 articles from databases (PubMed =39, Embase =62, Cochrane =2, clinicaltrials.gov =0, CNKI =180, other sources (from manually search) = 0). In Figure 1, the selection process was exhibited. In this process, we excluded 6 full-text articles (1 duplicate study [11]; 5 not case-control study [12–16]). In total, 4 articles [17–20] with 618 cases and 932 controls were included finally. In Tables 1 and 2, each study’s characteristics were exhibited. In the 4 articles, PCR–RFLP or Fluorescent CE-SSCP analysis were used as genotyping methods, and blood samples were utilized.

**Table 1.** Characteristics of studies included in the meta-analysis.

NO.	Study ID	Year	Country or Area	Ethnicity	Control Type	Genotyping Method	Case			Control			P for HWE*	Quality
	MPO -463G > A						AA	AG	GG	AA	AG	GG		
1	Buraczynska K [17]	2004	Poland	Caucasian	PB*	PCR-RFLP	5	28	62	4*	*37*	*74*	0.812	8
1.1	Diabetic nephropathy						4	6	27	*4*	*37*	*74*		
1.2	Other renal diseases						1	22	35	*4*	*37*	*74*		
2	Bouali H [18]	2007	USA	Caucasian and African American	PB	PCR-RFLP	4	22	16	9	101	167	0.178	8
2.1	Caucasian						1	2	4	4	69	130	0.132	
2.2	African American						3	20	12	5	32	37	0.583	
3	Doi K [19]	2007	Japan	Asian	PB	Fluorescent CE-SSCP analysis	3	102	326	*3*	*83*	*404*	0.569	8
3.1	Diabetic nephropathy						1	37	97	*3*	*83*	*404*		
3.2	Other renal diseases						2	65	229	*3*	*83*	*404*		
3.2.1	Chronic glomerulonephritis						1	56	167	*3*	*83*	*404*		
3.2.2	Hypertensive nephrosclerosis						1	9	62	*3*	*83*	*404*		
4	Debadwar S [20]	2016	India	Indian	NA*	PCR-RFLP	1	12	37	1	13	36	0.890	6

*HWE: Hardy–Weinberg equilibrium; PB: population-based; NA: not available.

*Control group shared with other studies were marked as italics.

**Table 2.** Characteristics of cases and controls.

Study ID	Case	Control
Buraczynska K [17]	37 ESRD* patients from diabetic nephropathy treated with peritoneal dialysis (33 of 37 have hypertension); 58 ESRD patients from other primary renal diseases treated with dialysis (49 of 58 have hypertension).	115 Healthy individuals (mainly blood donors and hospital employees) with normal blood urea level, serum creatinine, and blood pressure.
Bouali H [18]	7 Caucasian SLE* patients with lupus nephritis; 35 African American SLE patients with lupus nephritis. All patients were biopsy-confirmed Class III or IV lupus nephritis	Matched controls (203 Caucasian and 74 African American) were randomly selected from state driver's license registries.
Doi K [19]	431 ESRD patients treated with hemodialysis : (224 chronic glomerulonephritis, 135 diabetic nephropathy and 72 hypertensive nephrosclerosis.).	490 Healthy individuals from routine health checkups without urinary abnormality, renal dysfunction, or hyperglycemia.
Debadwar S [20]	50 patients with CKD* (stages 3 to 5).	50 Healthy controls.

*ESRD: end-stage renal disease; SLE: systemic lupus erythematosus; CKD: chronic kidney disease.

### 3.2. Overall analyses and subgroup analyses

In Table 3, we exhibit the summary results of every genetic model. Significantly increased risk of CKD was found in allelic comparison (A vs. G), heterozygote comparison (GA vs. GG) and dominant model (GA + AA vs. GG) of group ORD (other renal diseases), ORD plus, overall and overall plus. Other analyses did not show statistically significant changes of CKD risk.

**Table 3.** Summary of pooled ORs in the meta-analysis.

	Number	A vs. G		AA vs. GG		GA vs. GG		GA + AA vs. GG		AA vs. GG + GA	
	(cases/controls)	OR^a^(95%CI^a^)	I^2^(%)	OR(95%CI)	I^2^(%)	OR(95%CI)	I^2^(%)	OR(95%CI)	I^2^(%)	OR(95%CI)	I^2^(%)
Overall (1*, 2.1, 2.2, 3, 4)	618/932	1.306(1.048-1.626)**	0.0	1.609(0.728–3.555)	0.0	***1.339(1.038-1.728)***	0.0	***1.354(1.056-1.737)***	0.0	1.460(0.673-3.170)	0.0
Overall plus (1.1, 1.2, 2.1, 2.2, 3.1, 3.2.1, 3.2.2, 4)	618/2027	***1.303(1.076-1.577)***	0.0	1.588(0.788–3.202)	0.0	***1.329(1.066-1.656)***	44.4	***1.345(1.084-1.669)***	27.8	1.484(0.746-2.953)	0.0
DN^a^ (1.1, 3.1)	172/605	1.358(0.791-2.334)	**50.9**	2.243(0.675–7.461)	0.0	0.971(0.239-3.948)	**85.7**	1.179(0.439-3.164)	**78.1**	2.448(0.752-7.967)	0.0
ORD^a^ (1.2, 2.1, 2.2, 3.2)	396/882	***1.305(1.015-1.679)***	0.0	1.382(0.537–3.554)	0.0	***1.396(1.041-1.871)***	0.0	***1.390(1.042-1.853)***	0.0	1.175(0.465-2.968)	9.5
ORD plus (1.2, 2.1, 2.2, 3.2.1, 3.2.2)	396/1372	***1.287(1.013-1.635)***	0.0	1.391(0.559–3.460)	0.0	***1.362(1.033-1.797)***	15.8	***1.359(1.036-1.783)***	4.1	1.195(0.487-2.929)	0.0

^a^OR: Odds ratio; CI: confidence interval; DN: diabetic nephropathy; ORD: other renal diseases.

*NO of studies included in the meta-analysis.

**Results with statistical significant difference were marked as bold. Unstable results in sensitivity analyses were marked as italic. Less than three studies were included in DN, so that sensitivity analyses could not be performed.

### 3.3. Sensitivity analyses

In any comparison and any subgroup including more than two studies, sensitivity analyses were conducted. Because only two studies were included in DN (diabetic nephropathy), sensitivity analyses could not be done.

In group ORD, ORD plus, overall and overall plus, when study Doi K [19] was excluded, statistically different results were gained in allelic comparison (A vs. G), heterozygote comparison (GA vs. GG) and dominant model (GA + AA vs. GG). When study Bouali H [18] was excluded, statistically different results were gained in heterozygote comparison (GA vs. GG) overall, and in allelic comparison (A vs. G), heterozygote comparison (GA vs. GG) and dominant model (GA + AA vs. GG) of ORD and ORD plus. (Table 3 and Supplementary data)

Other results showed stability in sensitivity analyses. (Table 3 and Supplementary data)

### 3.4. Publication bias

The publication bias was evaluated by using Begg’s funnel plot and Egger’s test in every genetic model. In Begg’s funnel plot and Egger’s test, symmetry of funnel plot, *p* values of Begg’s test (P_B_) and *p* values of Egger’s test (P_E_) were used. We did not find significant publication bias. (Supplementary data)

### 3.5. Correlated component regression

Further statistics analysis was done in allelic comparison (A vs. G) by using a kind of logistic regression called Correlated Component Regression (CCR). CCR provides reliable predictions even with near multicollinear data. Near multicollinearity occurs when a large number of correlated predictors and relatively small sample size exists as well as situations involving a relatively small number of correlated predictors [21]. In our CCR Logistic, 10 rounds of 10-fold cross-validation was performed. In the goodness of fit statistics, AUC (area under curve) of cross-validation was 0.479 (SD =0.009, SE =1.62e-4).

## 4. Discussion

In group ORD, ORD plus, overall and overall plus, we found MPO -463G > A was not associated with CKD susceptibility in recessive model (AA vs. GG + GA) and homozygote comparison (AA vs. GG), and the results showed stability in sensitivity analyses and no publication bias.

**Figure 2.**
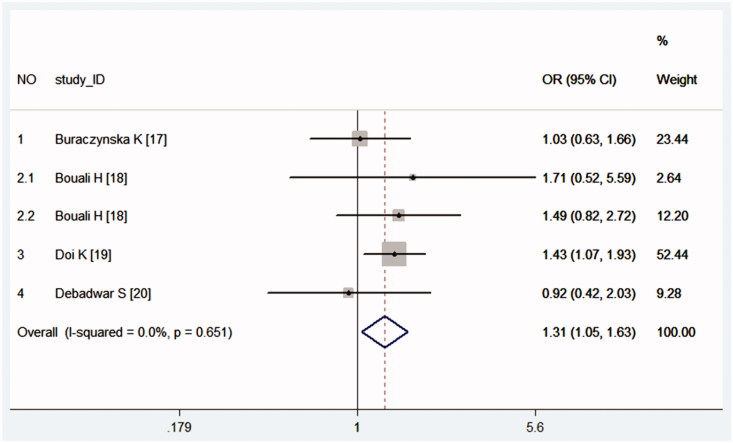
Forest plot with a fixed effects model for the association between chronic kidney disease and MPO -463G > A in allelic comparison (G vs. T). For each study, the estimate of OR and its 95% CI is plotted with a box and a horizontal line. Rhombus: pooled OR and its 95% CI.

In group ORD, ORD plus, overall and overall plus, we found MPO -463G > A was associated with increased risk of CKD in allelic comparison (A vs. G), heterozygote comparison (GA vs. GG) and dominant model (GA + AA vs. GG), however, the results lacked stability. Mostly, the stability was affected by study Doi K [19] and Bouali H [18]. The weight of study Doi K [19] in those meta-analysis is about 50% (such as Figure 2), which might shake the stability. When study Bouali H [18] was excluded, statistically different results were obtained in heterozygote comparison (GA vs. GG) overall, and in allelic comparison (A vs. G), heterozygote comparison (GA vs. GG) and dominant model (GA + AA vs. GG) of ORD and ORD plus. It seems study Bouali H [18] mostly affects group ORD. Glomerulonephritis, hypertensive nephrosclerosis, and diabetic nephropathy are the major pathogeny of CKD. Study by Bouali H [18] is about SLE patients with lupus nephritis, which might be different from the rest.

In group DN, we cannot conduct sensitivity analyses and publication bias analyses.

Moreover, our meta-analysis has several limitations. To date, only four eligible studies can be found and performed meta-analysis. Due to scanty data, subgroup analyses could not be performed well, and in some subgroups, sensitivity analyses and publication bias analyses could not be done. The controls were shared with each other in some studies, which were counted repeatedly. We might miss unpublished studies or studies written by other languages.

In Correlated Component Regression, AUC (area under curve) of cross-validation was 0.479 (SD =0.009, SE =1.62e-4). AUC＜0.5 might indicate a weak association between MPO -463G > A and CKD susceptibility.

In conclusion, our results suggested that: MPO -463G > A was not associated with CKD susceptibility in the recessive model and homozygote comparison. MPO -463G > A was associated with increased risk of CKD in allelic comparison, heterozygote comparison and dominant model, however, the results lacked stability. Owing to insufficient data, the association between MPO -463G > A and CKD cannot be fully confirmed, and the result should be explained carefully. Well-designed study with enough data are needed to perfect the current meta-analysis.

## Supplementary Material

Supplementary Data
