# Supplementary material for: Association between MPO-463G > A polymorphism and chronic kidney disease: a meta-analysis
Source: Ren Fail. 2018 Oct 3;40(1):541–6. doi: 10.1080/0886022X.2018.1499529 (PMC6171445; doi:10.1080/0886022X.2018.1499529)
Supplement: Supplementary Data [file IRNF_A_1499529_SM8274.docx]

**Supplementary data:** *Sensitivity analyses results and Publication bias results*

1：allelic comparison (A vs G)

2：homozygote comparison (AA vs GG)

3：heterozygote comparison (GA vs GG)

4：dominant model (GA+AA vs GG)

5：recessive model (AA vs GG+GA)

***Sensitivity analyses results*** ***and Publication bias results：***

Overall

1

------------------------------------------------------------------------------

Study ommited | e^coef. [95% Conf. Interval]

-------------------+----------------------------------------------------------

Buraczynska K [17]| 1.39283 1.0882804 1.7826063

Bouali H [18] | 1.2953145 1.0358626 1.6197511

Bouali H [18] | 1.2815966 1.0122843 1.622558

**Doi K [19] | 1.169734 .84390351 1.6213674**

Debadwar S [20] | 1.3467486 1.0714021 1.6928581

-------------------+----------------------------------------------------------

Combined | 1.307694 1.0498157 1.628918

Tests for Publication Bias

Begg's Test

adj. Kendall's Score (P-Q) = -2

Std. Dev. of Score = 4.08

Number of Studies = 5

z = -0.49

Pr > |z| = 0.624

z = 0.24 (continuity corrected)

Pr > |z| = 0.806 (continuity corrected)

Egger's test

------------------------------------------------------------------------------

Std_Eff | Coef. Std. Err. t P>|t| [95% Conf. Interval]

-------------+----------------------------------------------------------------

slope | .3609483 .2315081 1.56 0.217 -.3758138 1.09771

bias | -.4085641 .9238398 -0.44 0.688 -3.348635 2.531506

------------------------------------------------------------------------------

2

------------------------------------------------------------------------------

Study ommited | e^coef. [95% Conf. Interval]

-------------------+----------------------------------------------------------

Buraczynska K [17]| 1.8823724 .72263786 4.9033215

Bouali H [18] | 1.4526192 .63512785 3.3223271

Bouali H [18] | 1.7084948 .69329477 4.2102647

Doi K [19] | 1.9370892 .79096758 4.743955

Debadwar S [20] | 1.8299596 .81035132 4.1324695

-------------------+----------------------------------------------------------

Combined | 1.742481 .79687997 3.8101597

Tests for Publication Bias

Begg's Test

adj. Kendall's Score (P-Q) = 0

Std. Dev. of Score = 4.08

Number of Studies = 5

z = 0.00

Pr > |z| = 1.000

z = -0.24 (continuity corrected)

Pr > |z| = 1.000 (continuity corrected)

Egger's test

------------------------------------------------------------------------------

Std_Eff | Coef. Std. Err. t P>|t| [95% Conf. Interval]

-------------+----------------------------------------------------------------

slope | -.2169264 1.200639 -0.18 0.868 -4.037897 3.604044

bias | .8938608 1.345143 0.66 0.554 -3.386984 5.174706

------------------------------------------------------------------------------

3

------------------------------------------------------------------------------

Study ommited | e^coef. [95% Conf. Interval]

-------------------+----------------------------------------------------------

Buraczynska K [17]| 1.4649969 1.10293 1.9459222

Bouali H [18] | 1.3502951 1.0420247 1.7497636

**Bouali H [18] | 1.2926903 .98826259 1.6908948**

**Doi K [19] | 1.0825945 .71275506 1.6443388**

Debadwar S [20] | 1.3866329 1.0615913 1.8111968

-------------------+----------------------------------------------------------

Combined | 1.339574 1.0367379 1.73087

Tests for Publication Bias

Begg's Test

adj. Kendall's Score (P-Q) = -2

Std. Dev. of Score = 4.08

Number of Studies = 5

z = -0.49

Pr > |z| = 0.624

z = 0.24 (continuity corrected)

Pr > |z| = 0.806 (continuity corrected)

Egger's test

------------------------------------------------------------------------------

Std_Eff | Coef. Std. Err. t P>|t| [95% Conf. Interval]

-------------+----------------------------------------------------------------

slope | .4950931 .2722111 1.82 0.167 -.371204 1.36139

bias | -.7953116 .9310367 -0.85 0.456 -3.758286 2.167663

------------------------------------------------------------------------------

4

------------------------------------------------------------------------------

Study ommited | e^coef. [95% Conf. Interval]

-------------------+----------------------------------------------------------

Buraczynska K [17]| 1.4698172 1.1126518 1.9416341

Bouali H [18] | 1.3544829 1.0511684 1.7453187

Bouali H [18] | 1.3082149 1.006526 1.7003299

**Doi K [19] | 1.1381159 .76260387 1.6985328**

Debadwar S [20] | 1.4025417 1.0806239 1.8203588

-------------------+----------------------------------------------------------

Combined | 1.3539715 1.054388 1.7386756

Tests for Publication Bias

Begg's Test

adj. Kendall's Score (P-Q) = -2

Std. Dev. of Score = 4.08

Number of Studies = 5

z = -0.49

Pr > |z| = 0.624

z = 0.24 (continuity corrected)

Pr > |z| = 0.806 (continuity corrected)

Egger's test

------------------------------------------------------------------------------

Std_Eff | Coef. Std. Err. t P>|t| [95% Conf. Interval]

-------------+----------------------------------------------------------------

slope | .4363951 .2691484 1.62 0.203 -.4201551 1.292945

bias | -.5311939 .9433507 -0.56 0.613 -3.533357 2.470969

------------------------------------------------------------------------------

5

------------------------------------------------------------------------------

Study ommited | e^coef. [95% Conf. Interval]

-------------------+----------------------------------------------------------

Buraczynska K [17]| 1.6190135 .6367042 4.1168329

Bouali H [18] | 1.3060894 .58018689 2.9402068

Bouali H [18] | 1.7171716 .70263059 4.1966265

Doi K [19] | 1.7599944 .73558646 4.2110349

Debadwar S [20] | 1.6547296 .74575325 3.6716301

-------------------+----------------------------------------------------------

Combined | 1.5934307 .74033011 3.4295799

Tests for Publication Bias

Begg's Test

adj. Kendall's Score (P-Q) = -2

Std. Dev. of Score = 4.08

Number of Studies = 5

z = -0.49

Pr > |z| = 0.624

z = 0.24 (continuity corrected)

Pr > |z| = 0.806 (continuity corrected)

Egger's test

------------------------------------------------------------------------------

Std_Eff | Coef. Std. Err. t P>|t| [95% Conf. Interval]

-------------+----------------------------------------------------------------

slope | -.3941157 1.244232 -0.32 0.772 -4.353818 3.565587

bias | 1.016334 1.422738 0.71 0.527 -3.511453 5.544122

------------------------------------------------------------------------------

Overall plus

1

------------------------------------------------------------------------------

Study ommited | e^coef. [95% Conf. Interval]

-------------------+----------------------------------------------------------

Buraczynska K [17]| 1.3558038 1.109684 1.6565112

Buraczynska K [17]| 1.3548401 1.1043193 1.6621929

Bouali H [18] | 1.3083338 1.077179 1.5890927

Bouali H [18] | 1.2992087 1.0611636 1.590653

**Doi K [19] | 1.2249092 .98486784 1.5234557**

**Doi K [19] | 1.2509205 .99493645 1.5727658**

Doi K [19] | 1.376746 1.1263692 1.6827781

Debadwar S [20] | 1.347472 1.1056408 1.6421976

-------------------+----------------------------------------------------------

Combined | 1.3175169 1.0875216 1.596153

Tests for Publication Bias

Begg's Test

adj. Kendall's Score (P-Q) = -8

Std. Dev. of Score = 8.08

Number of Studies = 8

z = -0.99

Pr > |z| = 0.322

z = 0.87 (continuity corrected)

Pr > |z| = 0.386 (continuity corrected)

Egger's test

------------------------------------------------------------------------------

Std_Eff | Coef. Std. Err. t P>|t| [95% Conf. Interval]

-------------+----------------------------------------------------------------

slope | .6479017 .264436 2.45 0.050 .0008502 1.294953

bias | -1.422384 .9551409 -1.49 0.187 -3.75953 .9147614

------------------------------------------------------------------------------

2

------------------------------------------------------------------------------

Study ommited | e^coef. [95% Conf. Interval]

-------------------+----------------------------------------------------------

Buraczynska K [17]| 1.5486937 .68171046 3.5182858

Buraczynska K [17]| 2.042891 .96063983 4.3444

Bouali H [18] | 1.5346279 .72606127 3.2436419

Bouali H [18] | 1.7592056 .78865384 3.9241605

Doi K [19] | 1.8263929 .8603226 3.8772794

Doi K [19] | 1.9390356 .91326159 4.1169574

Doi K [19] | 1.7391527 .81937353 3.6914204

Debadwar S [20] | 1.8532612 .88514094 3.8802601

-------------------+----------------------------------------------------------

Combined | 1.777586 .86988588 3.6324443

Tests for Publication Bias

Begg's Test

adj. Kendall's Score (P-Q) = 2

Std. Dev. of Score = 8.08

Number of Studies = 8

z = 0.25

Pr > |z| = 0.805

z = 0.12 (continuity corrected)

Pr > |z| = 0.902 (continuity corrected)

Egger's test

------------------------------------------------------------------------------

Std_Eff | Coef. Std. Err. t P>|t| [95% Conf. Interval]

-------------+----------------------------------------------------------------

slope | 1.476961 1.265982 1.17 0.288 -1.620786 4.574709

bias | -.8958046 1.227546 -0.73 0.493 -3.899501 2.107892

------------------------------------------------------------------------------

.

3

------------------------------------------------------------------------------

Study ommited | e^coef. [95% Conf. Interval]

-------------------+----------------------------------------------------------

Buraczynska K [17]| 1.4546987 1.1552098 1.8318304

Buraczynska K [17]| 1.3797127 1.0871879 1.7509459

Bouali H [18] | 1.3738845 1.0957758 1.7225773

Bouali H [18] | 1.3310418 1.055095 1.679159

**Doi K [19] | 1.2304688 .94935469 1.5948238**

**Doi K [19] | 1.2444583 .94403845 1.6404803**

Doi K [19] | 1.4597272 1.1535727 1.8471341

Debadwar S [20] | 1.4026776 1.1129067 1.7678971

-------------------+----------------------------------------------------------

Combined | 1.3651229 1.0908863 1.7082996

Tests for Publication Bias

Begg's Test

adj. Kendall's Score (P-Q) = -14

Std. Dev. of Score = 8.08

Number of Studies = 8

z = -1.73

Pr > |z| = 0.083

z = 1.61 (continuity corrected)

Pr > |z| = 0.108 (continuity corrected)

Egger's test

------------------------------------------------------------------------------

Std_Eff | Coef. Std. Err. t P>|t| [95% Conf. Interval]

-------------+----------------------------------------------------------------

slope | .915427 .3213148 2.85 0.029 .1291981 1.701656

bias | -2.032289 .9928727 -2.05 0.087 -4.461761 .3971826

------------------------------------------------------------------------------

4

------------------------------------------------------------------------------

Study ommited | e^coef. [95% Conf. Interval]

-------------------+----------------------------------------------------------

Buraczynska K [17]| 1.4430386 1.1510541 1.8090899

Buraczynska K [17]| 1.3914143 1.104054 1.753568

Bouali H [18] | 1.3673083 1.097071 1.7041122

Bouali H [18] | 1.3331527 1.0637014 1.6708598

**Doi K [19] | 1.2416719 .96642727 1.5953078**

**Doi K [19] | 1.2638954 .96873629 1.648985**

Doi K [19] | 1.4538845 1.1562197 1.8281822

Debadwar S [20] | 1.4038036 1.1210852 1.7578188

-------------------+----------------------------------------------------------

Combined | 1.3666529 1.0990291 1.6994455

Tests for Publication Bias

Begg's Test

adj. Kendall's Score (P-Q) = -8

Std. Dev. of Score = 8.08

Number of Studies = 8

z = -0.99

Pr > |z| = 0.322

z = 0.87 (continuity corrected)

Pr > |z| = 0.386 (continuity corrected)

Egger's test

------------------------------------------------------------------------------

Std_Eff | Coef. Std. Err. t P>|t| [95% Conf. Interval]

-------------+----------------------------------------------------------------

slope | .786179 .3194617 2.46 0.049 .0044845 1.567874

bias | -1.621847 1.015757 -1.60 0.161 -4.107316 .8636217

------------------------------------------------------------------------------

5

------------------------------------------------------------------------------

Study ommited | e^coef. [95% Conf. Interval]

-------------------+----------------------------------------------------------

Buraczynska K [17]| 1.3743395 .61477344 3.072366

Buraczynska K [17]| 1.9559347 .93278328 4.1013606

Bouali H [18] | 1.4532082 .69594336 3.0344626

Bouali H [18] | 1.8384299 .82938493 4.0750975

Doi K [19] | 1.7627488 .84236568 3.688758

Doi K [19] | 1.8606607 .88907778 3.8939881

Doi K [19] | 1.6485682 .78795297 3.4491615

Debadwar S [20] | 1.7623781 .85320473 3.6403651

-------------------+----------------------------------------------------------

Combined | 1.7006659 .84264096 3.4323805

------------------------------------------------------------------------------

Tests for Publication Bias

Begg's Test

adj. Kendall's Score (P-Q) = 6

Std. Dev. of Score = 8.08

Number of Studies = 8

z = 0.74

Pr > |z| = 0.458

z = 0.62 (continuity corrected)

Pr > |z| = 0.536 (continuity corrected)

Egger's test

------------------------------------------------------------------------------

Std_Eff | Coef. Std. Err. t P>|t| [95% Conf. Interval]

-------------+----------------------------------------------------------------

slope | 1.369612 1.365395 1.00 0.355 -1.971389 4.710613

bias | -.8497899 1.347347 -0.63 0.551 -4.146629 2.447049

------------------------------------------------------------------------------

ORD

1

------------------------------------------------------------------------------

Study ommited | e^coef. [95% Conf. Interval]

-------------------+----------------------------------------------------------

Buraczynska K [17]| 1.3766025 1.0376747 1.8262319

**Bouali H [18] | 1.291165 .99781531 1.6707573**

**Bouali H [18] | 1.2716727 .96375156 1.6779755**

**Doi K [19] | 1.2900302 .877015 1.8975477**

-------------------+----------------------------------------------------------

Combined | 1.3076018 1.0164774 1.6821058

Tests for Publication Bias

Begg's Test

adj. Kendall's Score (P-Q) = 2

Std. Dev. of Score = 2.94

Number of Studies = 4

z = 0.68

Pr > |z| = 0.497

z = 0.34 (continuity corrected)

Pr > |z| = 0.734 (continuity corrected)

Egger's test

------------------------------------------------------------------------------

Std_Eff | Coef. Std. Err. t P>|t| [95% Conf. Interval]

-------------+----------------------------------------------------------------

slope | .1786782 .2068059 0.86 0.479 -.7111359 1.068492

bias | .3781838 .8047041 0.47 0.685 -3.084179 3.840546

2

------------------------------------------------------------------------------

Study ommited | e^coef. [95% Conf. Interval]

-------------------+----------------------------------------------------------

Buraczynska K [17]| 2.1064844 .72845997 6.0913115

Bouali H [18] | 1.2047846 .42371074 3.4257004

Bouali H [18] | 1.5136553 .4517494 5.0717331

Doi K [19] | 1.8573797 .5979973 5.7690219

-------------------+----------------------------------------------------------

Combined | 1.6308259 .62534488 4.2530019

Tests for Publication Bias

Begg's Test

adj. Kendall's Score (P-Q) = 0

Std. Dev. of Score = 2.94

Number of Studies = 4

z = 0.00

Pr > |z| = 1.000

z = -0.34 (continuity corrected)

Pr > |z| = 1.000 (continuity corrected)

Egger's test

------------------------------------------------------------------------------

Std_Eff | Coef. Std. Err. t P>|t| [95% Conf. Interval]

-------------+----------------------------------------------------------------

slope | -.5152262 3.40157 -0.15 0.894 -15.151 14.12055

bias | 1.041539 3.477667 0.30 0.793 -13.92165 16.00473

------------------------------------------------------------------------------

3

------------------------------------------------------------------------------

Study ommited | e^coef. [95% Conf. Interval]

-------------------+----------------------------------------------------------

Buraczynska K [17]| 1.4304718 1.0303797 1.985918

Bouali H [18] | 1.4111503 1.047013 1.9019297

**Bouali H [18] | 1.3358505 .97679579 1.8268881**

**Doi K [19] | 1.419896 .85947364 2.3457433**

-------------------+----------------------------------------------------------

Combined | 1.3946212 1.0392945 1.8714315

Tests for Publication Bias

Begg's Test

adj. Kendall's Score (P-Q) = -2

Std. Dev. of Score = 2.94

Number of Studies = 4

z = -0.68

Pr > |z| = 0.497

z = 0.34 (continuity corrected)

Pr > |z| = 0.734 (continuity corrected)

Egger's test

------------------------------------------------------------------------------

Std_Eff | Coef. Std. Err. t P>|t| [95% Conf. Interval]

-------------+----------------------------------------------------------------

slope | .3522842 .2073153 1.70 0.231 -.5397218 1.24429

bias | -.0741464 .6908487 -0.11 0.924 -3.046628 2.898335

------------------------------------------------------------------------------

4

------------------------------------------------------------------------------

Study ommited | e^coef. [95% Conf. Interval]

-------------------+----------------------------------------------------------

Buraczynska K [17]| 1.4423674 1.0456314 1.9896341

Bouali H [18] | 1.3898756 1.0362698 1.8641422

**Bouali H [18] | 1.3283408 .97695189 1.8061169**

**Doi K [19] | 1.413019 .86929764 2.296823**

-------------------+----------------------------------------------------------

Combined | 1.3878969 1.0402931 1.8516493

Tests for Publication Bias

Begg's Test

adj. Kendall's Score (P-Q) = 2

Std. Dev. of Score = 2.94

Number of Studies = 4

z = 0.68

Pr > |z| = 0.497

z = 0.34 (continuity corrected)

Pr > |z| = 0.734 (continuity corrected)

Egger's test

------------------------------------------------------------------------------

Std_Eff | Coef. Std. Err. t P>|t| [95% Conf. Interval]

-------------+----------------------------------------------------------------

slope | .2693969 .2010654 1.34 0.312 -.5957177 1.134512

bias | .2225947 .6834869 0.33 0.776 -2.718212 3.163401

5

------------------------------------------------------------------------------

Study ommited | e^coef. [95% Conf. Interval]

-------------------+----------------------------------------------------------

Buraczynska K [17]| 1.7614812 .62899779 4.9329524

Bouali H [18] | .99977477 .36101868 2.7686921

Bouali H [18] | 1.4755851 .44556147 4.8867589

Doi K [19] | 1.5309822 .51303216 4.568732

-------------------+----------------------------------------------------------

Combined | 1.4014518 .55087419 3.5653644

Tests for Publication Bias

Begg's Test

adj. Kendall's Score (P-Q) = 0

Std. Dev. of Score = 2.94

Number of Studies = 4

z = 0.00

Pr > |z| = 1.000

z = -0.34 (continuity corrected)

Pr > |z| = 1.000 (continuity corrected)

Egger's test

------------------------------------------------------------------------------

Std_Eff | Coef. Std. Err. t P>|t| [95% Conf. Interval]

-------------+----------------------------------------------------------------

slope | -1.041814 3.192958 -0.33 0.775 -14.78 12.69638

bias | 1.471534 3.351021 0.44 0.703 -12.94675 15.88982

------------------------------------------------------------------------------

ORD plus

1

------------------------------------------------------------------------------

Study ommited | e^coef. [95% Conf. Interval]

-------------------+----------------------------------------------------------

Buraczynska K [17]| 1.3583631 1.0389042 1.7760544

Bouali H [18] | 1.2836269 1.0031168 1.6425784

**Bouali H [18] | 1.2650572 .97200947 1.6464547**

**Doi K [19] | 1.1499612 .82496893 1.6029824**

Doi K [19] | 1.3951552 1.0758858 1.8091679

-------------------+----------------------------------------------------------

Combined | 1.2989487 1.0203461 1.653623

Tests for Publication Bias

Begg's Test

adj. Kendall's Score (P-Q) = -4

Std. Dev. of Score = 4.08

Number of Studies = 5

z = -0.98

Pr > |z| = 0.327

z = 0.73 (continuity corrected)

Pr > |z| = 0.462 (continuity corrected)

Egger's test

------------------------------------------------------------------------------

Std_Eff | Coef. Std. Err. t P>|t| [95% Conf. Interval]

-------------+----------------------------------------------------------------

slope | .4175134 .357116 1.17 0.327 -.718989 1.554016

bias | -.6040043 1.296615 -0.47 0.673 -4.730412 3.522403

------------------------------------------------------------------------------

2

------------------------------------------------------------------------------

Study ommited | e^coef. [95% Conf. Interval]

-------------------+----------------------------------------------------------

Buraczynska K [17]| 2.106888 .76072434 5.8351976

Bouali H [18] | 1.2581959 .4611086 3.4331539

Bouali H [18] | 1.5653395 .49735259 4.9266614

Doi K [19] | 1.9159208 .69449157 5.2855248

Doi K [19] | 1.5725945 .57047425 4.3350836

-------------------+----------------------------------------------------------

Combined | 1.6588059 .6568211 4.1893251

Tests for Publication Bias

Begg's Test

adj. Kendall's Score (P-Q) = 6

Std. Dev. of Score = 4.08

Number of Studies = 5

z = 1.47

Pr > |z| = 0.142

z = 1.22 (continuity corrected)

Pr > |z| = 0.221 (continuity corrected)

Egger's test

------------------------------------------------------------------------------

Std_Eff | Coef. Std. Err. t P>|t| [95% Conf. Interval]

-------------+----------------------------------------------------------------

slope | .2606953 2.889622 0.09 0.934 -8.93537 9.456761

bias | .2354999 2.733921 0.09 0.937 -8.465058 8.936058

------------------------------------------------------------------------------

3

------------------------------------------------------------------------------

Study ommited | e^coef. [95% Conf. Interval]

-------------------+----------------------------------------------------------

Buraczynska K [17]| 1.4117275 1.0340436 1.9273602

Bouali H [18] | 1.3971536 1.0499502 1.8591722

**Bouali H [18] | 1.3280384 .98540609 1.7898063**

**Doi K [19] | 1.1385793 .75179113 1.7243657**

Doi K [19] | 1.5503952 1.1428772 2.1032229

-------------------+----------------------------------------------------------

Combined | 1.382484 1.042935 1.8325801

------------------------------------------------------------------------------

Tests for Publication Bias

Begg's Test

adj. Kendall's Score (P-Q) = -4

Std. Dev. of Score = 4.08

Number of Studies = 5

z = -0.98

Pr > |z| = 0.327

z = 0.73 (continuity corrected)

Pr > |z| = 0.462 (continuity corrected)

Egger's test

------------------------------------------------------------------------------

Std_Eff | Coef. Std. Err. t P>|t| [95% Conf. Interval]

-------------+----------------------------------------------------------------

slope | .6252975 .389769 1.60 0.207 -.6151215 1.865717

bias | -1.031 1.212169 -0.85 0.458 -4.888662 2.826663

------------------------------------------------------------------------------

4

------------------------------------------------------------------------------

Study ommited | e^coef. [95% Conf. Interval]

-------------------+----------------------------------------------------------

Buraczynska K [17]| 1.4186745 1.0462671 1.9236362

Bouali H [18] | 1.3749795 1.0388157 1.8199269

**Bouali H [18] | 1.3185858 .98450045 1.7660414**

**Doi K [19] | 1.157163 .77532558 1.7270501**

Doi K [19] | 1.5282284 1.1327961 2.0616967

-------------------+----------------------------------------------------------

Combined | 1.373673 1.0426407 1.8098063

Tests for Publication Bias

Begg's Test

adj. Kendall's Score (P-Q) = -2

Std. Dev. of Score = 4.08

Number of Studies = 5

z = -0.49

Pr > |z| = 0.624

z = 0.24 (continuity corrected)

Pr > |z| = 0.806 (continuity corrected)

Egger's test

------------------------------------------------------------------------------

Std_Eff | Coef. Std. Err. t P>|t| [95% Conf. Interval]

-------------+----------------------------------------------------------------

slope | .5165051 .3967626 1.30 0.284 -.7461705 1.779181

bias | -.6897492 1.261268 -0.55 0.623 -4.703666 3.324167

------------------------------------------------------------------------------

5

------------------------------------------------------------------------------

Study ommited | e^coef. [95% Conf. Interval]

-------------------+----------------------------------------------------------

Buraczynska K [17]| 1.802614 .66965633 4.8523654

Bouali H [18] | 1.0663227 .40009322 2.8419478

Bouali H [18] | 1.5481136 .49685274 4.8236741

Doi K [19] | 1.6504717 .61599173 4.4222296

Doi K [19] | 1.3308191 .49701887 3.563405

-------------------+----------------------------------------------------------

Combined | 1.4493305 .58692079 3.5789477

Tests for Publication Bias

Begg's Test

adj. Kendall's Score (P-Q) = 6

Std. Dev. of Score = 4.08

Number of Studies = 5

z = 1.47

Pr > |z| = 0.142

z = 1.22 (continuity corrected)

Pr > |z| = 0.221 (continuity corrected)

Egger's test

------------------------------------------------------------------------------

Std_Eff | Coef. Std. Err. t P>|t| [95% Conf. Interval]

-------------+----------------------------------------------------------------

slope | -.4715362 2.655821 -0.18 0.870 -8.923543 7.980471

bias | .8317385 2.57519 0.32 0.768 -7.363665 9.027142

------------------------------------------------------------------------------
